# Erratum

**DOI:** 10.1097/MD.0000000000022388

**Published:** 2020-09-11

Medicine is issuing corrections regarding two articles. Both articles were investigated after reader concerns were raised. Medicine previously issued a Letter of Concern^[1,2]^ documenting the concerns and published the initial Letter to the Editor on the Correspondence Blog.

In response to the reader concerns and the internal investigation, the authors of “A randomized controlled trial on the effectiveness of 8-week high intensity interval exercise on interahepatic triglycerides, visceral lipids, and health-related quality of life in diabetic obese patients with nonalcoholic fatty liver disease”^[3]^ which appeared in Volume 98, Issue 12 of *Medicine*, have updated the clinical trial registration information to reflect the trial start and completion dates for all interventional trial arms and the number of participants. The clinical trial registration number (NCT03774511) has been added to the manuscript details.

Authors also sought additional statistical advice to the concerns raised regarding published article **“**Effects of high-intensity interval and moderate-intensity continuous aerobic exercise on diabetic obese patients with nonalcoholic fatty liver disease: a comparative randomized controlled trial”^4^ which appeared in Volume 99, Issue 10 of *Medicine*. Authors followed the recommendation to conduct a One Way ANOVA test between the three groups post-intervention. Below are the results of the second opinion and an updated conclusion.

“Using one way ANOVA test to assess the differences between the three groups post-intervention, there were significant differences in visceral adipose fat, triglycerides, total cholesterol, TGs, LDL, ALT, HbA1c, and HOMA-IR (p < 0.05) while no significant differences were observed in BMI, IHTG, and HDL (p > 0.05).”

**Table:**
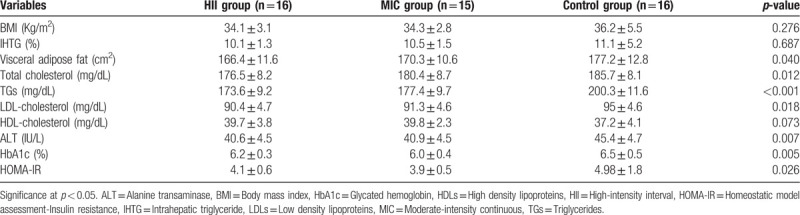
Table 2 The differences between HII, MIC, and control groups post-intervention (Article 3).

It should also be noted that randomization for experimental intervention arms in the above listed articles occurred all at once, but baseline data collection occurred six months apart creating a potential time confound.

## Correction

The name Foote was originally written incorrectly as Goote and has been corrected.
